# Bis(2-methyl­benzoato-κ^2^ *O*,*O*′)(1,10′-phenanthroline-κ^2^ *N*,*N*′)copper(II)

**DOI:** 10.1107/S1600536811018162

**Published:** 2011-05-20

**Authors:** Sheng-Liang Ni, Feng Zhou, Jin-Li Qi

**Affiliations:** a Department of Chemistry, Huzhou Teachers College, Huzhou, Zhejiang, 313000, People’s Republic of China

## Abstract

In the title compound, [Cu(C_8_H_7_O_2_)_2_(C_12_H_8_N_2_)], the Cu^II^ atom assumes a distorted octa­hedral coordination geometry, chelated by two N atoms from the 1,10′-phenanthroline ligand and four O atoms from two 2-methyl­benzoate anions. A significant Jahn–Teller distortion is observed with two axial Cu—O distances significantly longer than those in the equatorial CuO_2_N_2_ plane. In the crystal, π–π stacking inter­actions, with centroid–centroid distances of 3.547 (3) or 3.728 (3) Å between the phenanthroline rings, form layers parallel to (011).

## Related literature

For Jahn–Teller distortions in copper complexes, see: Yang & Vittal (2003 ▶); Su *et al.* (2005 ▶); Liu *et al.* (2010 ▶). For phenanthroline complexes, see: Wang *et al.* (1996 ▶); Wall *et al.* (1999 ▶); Naing *et al.* (1995 ▶). For related structures, see: Cano *et al.* (1997 ▶); Rodrigues *et al.* (1999 ▶) Xu & Xu (2004 ▶).
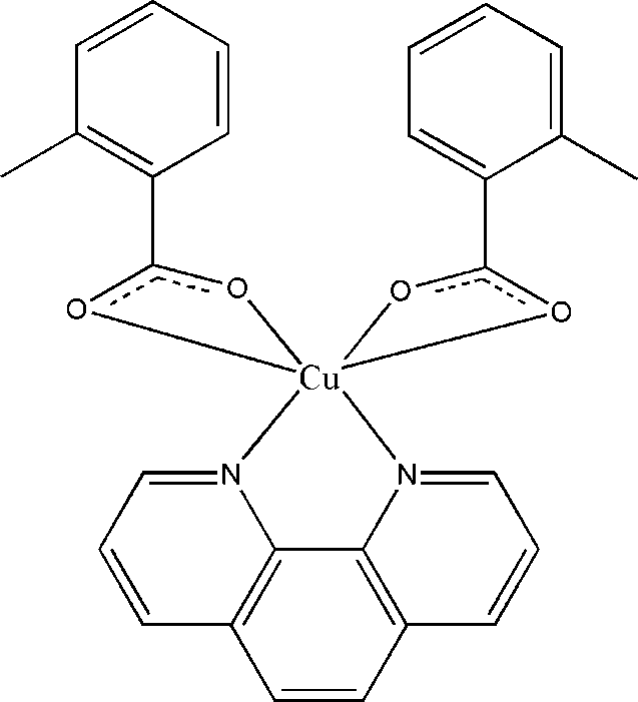


## Experimental

### 

#### Crystal data

[Cu(C_8_H_7_O_2_)_2_(C_12_H_8_N_2_)]
*M* *_r_* = 514.02
Monoclinic, 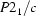

*a* = 16.245 (3) Å
*b* = 10.136 (2) Å
*c* = 14.048 (3) Å
β = 99.15 (3)°
*V* = 2283.7 (8) Å^3^
*Z* = 4
Mo *K*α radiation
μ = 1.00 mm^−1^
*T* = 293 K
0.15 × 0.10 × 0.10 mm

#### Data collection

Rigaku R-AXIS RAPID diffractometer
Absorption correction: multi-scan (*ABSCOR*; Higashi, 1995 ▶) *T*_min_ = 0.866, *T*_max_ = 0.900
17441 measured reflections
4021 independent reflections
2509 reflections with *I* > 2σ(*I*)
*R*_int_ = 0.063

#### Refinement

*R*[*F* ^2^ > 2σ(*F* ^2^)] = 0.045
*wR*(*F* ^2^) = 0.150
*S* = 1.13
4021 reflections
319 parameters
H-atom parameters constrained
Δρ_max_ = 0.74 e Å^−3^
Δρ_min_ = −1.04 e Å^−3^

### 

Data collection: *RAPID-AUTO* (Rigaku, 1998 ▶); cell refinement: *RAPID-AUTO*; data reduction: *CrystalStructure* (Rigaku/MSC, 2004 ▶); program(s) used to solve structure: *SHELXS97* (Sheldrick, 2008 ▶); program(s) used to refine structure: *SHELXL97* (Sheldrick, 2008 ▶); molecular graphics: *SHELXTL* (Sheldrick, 2008 ▶); software used to prepare material for publication: *SHELXTL*.

## Supplementary Material

Crystal structure: contains datablocks global, I. DOI: 10.1107/S1600536811018162/sj5144sup1.cif

Structure factors: contains datablocks I. DOI: 10.1107/S1600536811018162/sj5144Isup2.hkl

Additional supplementary materials:  crystallographic information; 3D view; checkCIF report

## Figures and Tables

**Table 1:** Selected bond lengths (Å)

Cu—O3	1.919 (4)
Cu—O1	1.927 (4)
Cu—N2	2.010 (4)
Cu—N1	2.025 (4)

**Fig. 1.**
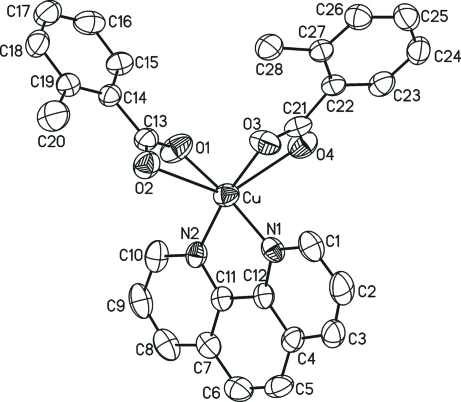
The structure of the title compound with dispalcement ellipsoids drawn at the 45% probability level.

**Fig. 2.**
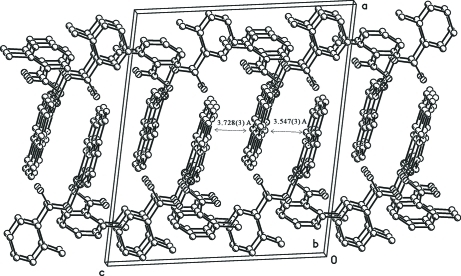
Crystal packing showing the two-dimensional layer structure linked through π–π stacking interactions.

## supplementary crystallographic information

### Comment

The Jahn-Teller distortion of copper(II) complexes is well known. Most copper(II) complexes display an elongated distortion, and the coordinate bonds in the axial direction are usually longer than those in the equatorial coordination plane by 0.2–0.6 Å (Yang  & Vittal, 2003; Su *et al.*, 2005; Liu *et al.*, 2010). Metal-phenanthroline complexes and their derivatives have also attracted much attention (Wang *et al.*, 1996; Wall *et al.*, 1999; Naing *et al.*, 1995). In the title copper(II) phenanthroline complex, (I), a pair of long Cu-O bonds is observed.

The molecular structure of the title complex is shown in Fig. 1. The Cu^II^ ion binds to a phenanthroline molecule and two 2-methylbenzoate anions in a distorted octahedral geometry. Two N atoms from phen and two O atoms from carboxyl groups form a tetrahedrally distorted equatorial coordination plane, with a dihedral angle of 7.3 (2) ° between the Cu/O1/O3 and Cu/N1/N2 planes. The bond lengths in the equatorial plane are normal (Table 1). In the axial direction, the Cu-O distances (Cu-O2 2.609 (4) Å, Cu-O4 2.666 (4) Å) are longer than the Cu-O distances(Cu-O1 1.927 (4) Å, Cu-O3 1.919 (4) Å) equatorial plane.

The Cu-O-C angles of (Cu-O1-C13 105.9 (3) °, Cu-O3-C21 108.2 (3) °) are similar to values found in copper(II) complexes with a chelating benzoate ligand, for example, 106.1 (4) ° (Cano *et al.*, 1997) and 104.5 (1) ° (Xu & Xu, 2004), but are much smaller than those in copper(II) complexes with a monodentate benzoate ligand, for example, 131.8 (1) ° (Rodrigues *et al.*, 1999). This suggests the existence of a bonding interaction between atoms Cu and O2, Cu and O4. Besides the elongated Jahn-Teller distortion, the smaller O2-Cu-O4 angle of 132.1 (1) Å is also a possible reason for the larger differences within the same carboxylate group.

In the crystal structure two-dimensional layers form parallel to (011) through  π–π packing interactions with centroid to centroid distances 3.547 (3) Å and 3.728 (3) Å between the phenanthroline rings, as shown in Fig. 2.

### Experimental

Freshly prepared CuCO_3_ was essential for an optimal synthesis. 1.0 cm^3^ (1 *M*) aqueous Na_2_CO_3_ was added dropwise to a stirred aqueous solution of (0.2490 g, 1.0 mmol)CuSO_4_.5H_2_O in 4 cm^3^ of doubly distilled water. This produced a blue  precipitate, of Cu(OH)_2-2x_(CO_3_)*_x_*.yH_2_O,  which was centrifuged and washed with doubly distilled water until no SO_4_^-2^ anions were detected in the supernatant liquid. The fresh blue precipitate was subsequently added to a stirred solution of 2-methylbenzoic acid (0.2725 g, 2.0 mmol) and 1,10'-phenanthroline (0.1982 g, 1.0 mmol) in 20 cm^3^ C_2_H_5_OH-H_2_O (1:1, *v*/*v*). The mixture was stirred for 30 min and filtered. The insoluble solid was then filtered off, and the resulting blue filtrate (pH = 5.20) was allowed to stand at room temperature. Blue block-like crystals were grown by slow evaporation over a week. Yield: 45% based on the initial CuSO_4_.5 H_2_O.

### Refinement

All H-atoms bonded to C were positioned geometrically and refined using a riding model with d(C-H) = 0.093 Å, *U*_iso_(H) = 1.2 *U*_eq_(C) for aromatic, and 0.96 Å, *U*_iso_(H) = 1.5 *U*_eq_(C) for CH_3_ atoms. H atoms attached to O atoms were found in a difference Fourier synthesis and were refined using a riding model, with the O-H distances fixed as initially found and with *U*_iso_(H) values set at 1.5 *U*eq(O).

### Crystal data

**Table:**

[Cu(C_8_H_7_O_2_)_2_(C_12_H_8_N_2_)]	*F*(000) = 1060
*M**_r_* = 514.02	*D*_x_ = 1.495 Mg m^−^^3^
Monoclinic, *P*2_1_/*c*	Mo *K*α radiation, λ = 0.71073 Å
Hall symbol: -P 2ybc	Cell parameters from 25 reflections
*a* = 16.245 (3) Å	θ = 3.0–25.0°
*b* = 10.136 (2) Å	µ = 1.00 mm^−^^1^
*c* = 14.048 (3) Å	*T* = 293 K
β = 99.15 (3)°	Plate, blue
*V* = 2283.7 (8) Å^3^	0.15 × 0.10 × 0.10 mm
*Z* = 4	

### Data collection

**Table:**

Rigaku R-AXIS RAPID diffractometer	4021 independent reflections
Radiation source: fine-focus sealed tube	2509 reflections with *I* > 2σ(*I*)
graphite	*R*_int_ = 0.063
ω scans	θ_max_ = 25.0°, θ_min_ = 3.0°
Absorption correction: multi-scan (*ABSCOR*; Higashi, 1995)	*h* = −19→19
*T*_min_ = 0.866, *T*_max_ = 0.900	*k* = −12→12
17441 measured reflections	*l* = −16→16

### Refinement

**Table:**

Refinement on *F*^2^	Secondary atom site location: difference Fourier map
Least-squares matrix: full	Hydrogen site location: inferred from neighbouring sites
*R*[*F*^2^ > 2σ(*F*^2^)] = 0.045	H-atom parameters constrained
*wR*(*F*^2^) = 0.150	*w* = 1/[σ^2^(*F*_o_^2^) + (0.0447*P*)^2^ + 4.7675*P*] where *P* = (*F*_o_^2^ + 2*F*_c_^2^)/3
*S* = 1.13	(Δ/σ)_max_ < 0.001
4021 reflections	Δρ_max_ = 0.74 e Å^−^^3^
319 parameters	Δρ_min_ = −1.04 e Å^−^^3^
0 restraints	Extinction correction: *SHELXL97* (Sheldrick, 2008), Fc^*^=kFc[1+0.001xFc^2^λ^3^/sin(2θ)]^-1/4^
Primary atom site location: structure-invariant direct methods	Extinction coefficient: 0.0013 (5)

### Special details

**Table:**

Geometry. All e.s.d.'s (except the e.s.d. in the dihedral angle between two l.s. planes) are estimated using the full covariance matrix. The cell e.s.d.'s are taken into account individually in the estimation of e.s.d.'s in distances, angles and torsion angles; correlations between e.s.d.'s in cell parameters are only used when they are defined by crystal symmetry. An approximate (isotropic) treatment of cell e.s.d.'s is used for estimating e.s.d.'s involving l.s. planes.
Refinement. Refinement of *F*^2^ against ALL reflections. The weighted *R*-factor *wR* and goodness of fit *S* are based on *F*^2^, conventional *R*-factors *R* are based on *F*, with *F* set to zero for negative *F*^2^. The threshold expression of *F*^2^ > σ(*F*^2^) is used only for calculating *R*-factors(gt) *etc*. and is not relevant to the choice of reflections for refinement. *R*-factors based on *F*^2^ are statistically about twice as large as those based on *F*, and *R*- factors based on ALL data will be even larger.

### Fractional atomic coordinates and isotropic or equivalent isotropic displacement parameters (Å^2^)

**Table:**

	*x*	*y*	*z*	*U*_iso_*/*U*_eq_	
Cu	−0.33221 (4)	0.96309 (7)	−0.17022 (4)	0.0480 (2)	
O1	−0.2996 (2)	1.1433 (4)	−0.1871 (3)	0.0703 (12)	
O2	−0.2404 (2)	1.1033 (4)	−0.0382 (3)	0.0606 (10)	
O3	−0.2325 (2)	0.8925 (4)	−0.2077 (3)	0.0635 (11)	
O4	−0.3140 (2)	0.8977 (4)	−0.3495 (3)	0.0609 (10)	
N1	−0.3735 (2)	0.7809 (4)	−0.1419 (3)	0.0450 (10)	
N2	−0.4462 (2)	1.0137 (4)	−0.1442 (3)	0.0423 (9)	
C1	−0.3348 (4)	0.6641 (6)	−0.1404 (4)	0.0584 (15)	
H1	−0.2794	0.6613	−0.1496	0.070*	
C2	−0.3756 (5)	0.5461 (6)	−0.1255 (4)	0.0698 (17)	
H2	−0.3471	0.4665	−0.1251	0.084*	
C3	−0.4558 (4)	0.5468 (6)	−0.1115 (4)	0.0607 (15)	
H3	−0.4825	0.4678	−0.1024	0.073*	
C4	−0.4990 (3)	0.6670 (5)	−0.1108 (3)	0.0478 (13)	
C5	−0.5834 (3)	0.6805 (6)	−0.0943 (4)	0.0587 (15)	
H5	−0.6141	0.6053	−0.0854	0.070*	
C6	−0.6191 (3)	0.7998 (7)	−0.0914 (4)	0.0588 (15)	
H6	−0.6734	0.8055	−0.0785	0.071*	
C7	−0.5753 (3)	0.9185 (6)	−0.1076 (3)	0.0482 (13)	
C8	−0.6084 (4)	1.0457 (6)	−0.1077 (4)	0.0606 (16)	
H8	−0.6627	1.0579	−0.0962	0.073*	
C9	−0.5615 (4)	1.1519 (6)	−0.1244 (4)	0.0623 (16)	
H9	−0.5833	1.2366	−0.1235	0.075*	
C10	−0.4806 (4)	1.1328 (5)	−0.1431 (4)	0.0565 (14)	
H10	−0.4494	1.2061	−0.1552	0.068*	
C11	−0.4933 (3)	0.9076 (5)	−0.1268 (3)	0.0395 (11)	
C12	−0.4540 (3)	0.7812 (5)	−0.1262 (3)	0.0406 (11)	
C13	−0.2490 (3)	1.1732 (5)	−0.1108 (4)	0.0476 (12)	
C14	−0.2009 (3)	1.3008 (5)	−0.1152 (3)	0.0416 (11)	
C15	−0.2043 (3)	1.3578 (5)	−0.2067 (4)	0.0509 (13)	
H15	−0.2348	1.3161	−0.2599	0.061*	
C16	−0.1637 (3)	1.4737 (6)	−0.2196 (4)	0.0619 (15)	
H16	−0.1660	1.5092	−0.2811	0.074*	
C17	−0.1197 (4)	1.5370 (6)	−0.1411 (5)	0.0667 (16)	
H17	−0.0928	1.6163	−0.1490	0.080*	
C18	−0.1157 (3)	1.4830 (6)	−0.0517 (5)	0.0604 (15)	
H18	−0.0859	1.5272	0.0008	0.072*	
C19	−0.1545 (3)	1.3636 (5)	−0.0356 (4)	0.0496 (13)	
C20	−0.1458 (4)	1.3130 (7)	0.0666 (4)	0.080 (2)	
H20A	−0.1327	1.2206	0.0676	0.120*	
H20B	−0.1973	1.3262	0.0907	0.120*	
H20C	−0.1019	1.3600	0.1063	0.120*	
C21	−0.2458 (3)	0.8741 (5)	−0.2988 (4)	0.0494 (13)	
C22	−0.1747 (3)	0.8162 (5)	−0.3429 (3)	0.0425 (11)	
C23	−0.1959 (3)	0.7396 (5)	−0.4251 (3)	0.0531 (14)	
H23	−0.2517	0.7325	−0.4527	0.064*	
C24	−0.1363 (4)	0.6736 (6)	−0.4669 (4)	0.0615 (15)	
H24	−0.1517	0.6217	−0.5214	0.074*	
C25	−0.0538 (4)	0.6861 (6)	−0.4265 (4)	0.0612 (15)	
H25	−0.0130	0.6410	−0.4528	0.073*	
C26	−0.0319 (3)	0.7651 (6)	−0.3470 (4)	0.0555 (14)	
H26	0.0243	0.7750	−0.3220	0.067*	
C27	−0.0903 (3)	0.8306 (5)	−0.3028 (3)	0.0430 (12)	
C28	−0.0598 (3)	0.9171 (6)	−0.2164 (4)	0.0629 (16)	
H18A	−0.0909	0.9981	−0.2215	0.094*	
H28B	−0.0017	0.9361	−0.2144	0.094*	
H28C	−0.0676	0.8720	−0.1584	0.094*	

### Atomic displacement parameters (Å^2^)

**Table:**

	*U*^11^	*U*^22^	*U*^33^	*U*^12^	*U*^13^	*U*^23^
Cu	0.0409 (4)	0.0540 (4)	0.0480 (4)	−0.0059 (3)	0.0037 (3)	0.0006 (3)
O1	0.067 (2)	0.074 (3)	0.063 (2)	−0.024 (2)	−0.011 (2)	0.009 (2)
O2	0.075 (3)	0.049 (2)	0.057 (2)	−0.006 (2)	0.0108 (19)	0.0059 (19)
O3	0.049 (2)	0.094 (3)	0.048 (2)	−0.005 (2)	0.0121 (17)	0.000 (2)
O4	0.045 (2)	0.060 (2)	0.074 (3)	−0.0035 (18)	−0.0034 (18)	0.005 (2)
N1	0.040 (2)	0.053 (3)	0.041 (2)	0.010 (2)	0.0046 (17)	−0.003 (2)
N2	0.051 (2)	0.035 (2)	0.039 (2)	0.0028 (19)	0.0003 (18)	0.0003 (18)
C1	0.059 (3)	0.059 (4)	0.056 (3)	0.022 (3)	0.005 (3)	−0.002 (3)
C2	0.105 (5)	0.041 (3)	0.065 (4)	0.018 (4)	0.019 (4)	0.004 (3)
C3	0.091 (5)	0.044 (3)	0.050 (3)	−0.007 (3)	0.019 (3)	−0.001 (3)
C4	0.056 (3)	0.047 (3)	0.039 (3)	−0.009 (3)	0.001 (2)	−0.003 (2)
C5	0.054 (3)	0.075 (4)	0.047 (3)	−0.027 (3)	0.005 (3)	−0.001 (3)
C6	0.041 (3)	0.084 (5)	0.050 (3)	−0.007 (3)	0.004 (2)	−0.007 (3)
C7	0.040 (3)	0.062 (4)	0.039 (3)	0.006 (3)	−0.002 (2)	−0.005 (2)
C8	0.050 (3)	0.078 (4)	0.050 (3)	0.025 (3)	−0.002 (2)	−0.009 (3)
C9	0.070 (4)	0.059 (4)	0.053 (3)	0.031 (3)	−0.004 (3)	−0.009 (3)
C10	0.081 (4)	0.040 (3)	0.044 (3)	0.006 (3)	−0.004 (3)	0.003 (2)
C11	0.039 (3)	0.044 (3)	0.033 (2)	0.002 (2)	−0.003 (2)	−0.002 (2)
C12	0.041 (3)	0.042 (3)	0.035 (2)	0.005 (2)	−0.003 (2)	−0.005 (2)
C13	0.041 (3)	0.042 (3)	0.061 (3)	0.001 (2)	0.013 (3)	0.000 (3)
C14	0.036 (2)	0.039 (3)	0.050 (3)	0.000 (2)	0.007 (2)	−0.001 (2)
C15	0.043 (3)	0.054 (3)	0.056 (3)	−0.003 (2)	0.006 (2)	0.005 (3)
C16	0.057 (3)	0.061 (4)	0.071 (4)	0.003 (3)	0.021 (3)	0.021 (3)
C17	0.060 (4)	0.047 (3)	0.096 (5)	−0.006 (3)	0.023 (3)	0.000 (4)
C18	0.049 (3)	0.053 (4)	0.079 (4)	−0.007 (3)	0.008 (3)	−0.018 (3)
C19	0.046 (3)	0.053 (3)	0.051 (3)	−0.005 (3)	0.011 (2)	−0.006 (3)
C20	0.086 (5)	0.098 (5)	0.053 (4)	−0.023 (4)	0.002 (3)	−0.003 (3)
C21	0.039 (3)	0.053 (3)	0.057 (3)	−0.013 (2)	0.007 (2)	0.006 (3)
C22	0.044 (3)	0.042 (3)	0.043 (3)	−0.005 (2)	0.009 (2)	0.007 (2)
C23	0.058 (3)	0.056 (3)	0.043 (3)	−0.015 (3)	0.002 (2)	0.003 (3)
C24	0.088 (4)	0.050 (3)	0.049 (3)	−0.014 (3)	0.017 (3)	−0.007 (3)
C25	0.070 (4)	0.054 (4)	0.063 (4)	0.006 (3)	0.022 (3)	0.000 (3)
C26	0.051 (3)	0.062 (4)	0.053 (3)	−0.001 (3)	0.007 (3)	0.002 (3)
C27	0.042 (3)	0.041 (3)	0.045 (3)	−0.002 (2)	0.007 (2)	0.005 (2)
C28	0.048 (3)	0.079 (4)	0.061 (3)	−0.014 (3)	0.003 (3)	−0.013 (3)

### Geometric parameters (Å, °)

**Table:**

Cu—O3	1.919 (4)	C11—C12	1.431 (6)
Cu—O1	1.927 (4)	C13—C14	1.518 (7)
Cu—N2	2.010 (4)	C14—C19	1.399 (7)
Cu—N1	2.025 (4)	C14—C15	1.402 (7)
O1—C13	1.279 (6)	C15—C16	1.374 (7)
O2—C13	1.230 (6)	C15—H15	0.9300
O3—C21	1.277 (6)	C16—C17	1.374 (8)
O4—C21	1.240 (6)	C16—H16	0.9300
N1—C1	1.339 (6)	C17—C18	1.362 (8)
N1—C12	1.361 (6)	C17—H17	0.9300
N2—C10	1.332 (6)	C18—C19	1.399 (7)
N2—C11	1.364 (6)	C18—H18	0.9300
C1—C2	1.399 (8)	C19—C20	1.510 (7)
C1—H1	0.9300	C20—H20A	0.9600
C2—C3	1.348 (8)	C20—H20B	0.9600
C2—H2	0.9300	C20—H20C	0.9600
C3—C4	1.406 (7)	C21—C22	1.515 (7)
C3—H3	0.9300	C22—C23	1.388 (7)
C4—C12	1.404 (7)	C22—C27	1.404 (6)
C4—C5	1.434 (7)	C23—C24	1.382 (8)
C5—C6	1.345 (8)	C23—H23	0.9300
C5—H5	0.9300	C24—C25	1.375 (8)
C6—C7	1.435 (8)	C24—H24	0.9300
C6—H6	0.9300	C25—C26	1.374 (7)
C7—C8	1.398 (7)	C25—H25	0.9300
C7—C11	1.404 (7)	C26—C27	1.383 (7)
C8—C9	1.360 (8)	C26—H26	0.9300
C8—H8	0.9300	C27—C28	1.516 (7)
C9—C10	1.393 (8)	C28—H18A	0.9600
C9—H9	0.9300	C28—H28B	0.9600
C10—H10	0.9300	C28—H28C	0.9600
			
O3—Cu—O1	93.39 (18)	O1—C13—C14	115.7 (5)
O3—Cu—N2	170.71 (16)	C19—C14—C15	118.9 (5)
O1—Cu—N2	93.45 (17)	C19—C14—C13	124.8 (4)
O3—Cu—N1	91.94 (17)	C15—C14—C13	116.3 (4)
O1—Cu—N1	173.96 (17)	C16—C15—C14	121.6 (5)
N2—Cu—N1	81.58 (15)	C16—C15—H15	119.2
C13—O1—Cu	105.9 (3)	C14—C15—H15	119.2
C21—O3—Cu	108.2 (3)	C15—C16—C17	119.5 (5)
C1—N1—C12	117.4 (5)	C15—C16—H16	120.3
C1—N1—Cu	129.8 (4)	C17—C16—H16	120.3
C12—N1—Cu	112.7 (3)	C18—C17—C16	119.7 (6)
C10—N2—C11	117.7 (4)	C18—C17—H17	120.2
C10—N2—Cu	129.3 (4)	C16—C17—H17	120.2
C11—N2—Cu	113.0 (3)	C17—C18—C19	122.7 (5)
N1—C1—C2	121.7 (5)	C17—C18—H18	118.7
N1—C1—H1	119.1	C19—C18—H18	118.7
C2—C1—H1	119.1	C14—C19—C18	117.6 (5)
C3—C2—C1	120.6 (6)	C14—C19—C20	124.2 (5)
C3—C2—H2	119.7	C18—C19—C20	118.1 (5)
C1—C2—H2	119.7	C19—C20—H20A	109.5
C2—C3—C4	120.0 (5)	C19—C20—H20B	109.5
C2—C3—H3	120.0	H20A—C20—H20B	109.5
C4—C3—H3	120.0	C19—C20—H20C	109.5
C12—C4—C3	116.2 (5)	H20A—C20—H20C	109.5
C12—C4—C5	118.7 (5)	H20B—C20—H20C	109.5
C3—C4—C5	125.1 (5)	O4—C21—O3	122.7 (5)
C6—C5—C4	121.3 (5)	O4—C21—C22	120.6 (5)
C6—C5—H5	119.4	O3—C21—C22	116.6 (4)
C4—C5—H5	119.4	C23—C22—C27	119.3 (5)
C5—C6—C7	121.4 (5)	C23—C22—C21	116.9 (4)
C5—C6—H6	119.3	C27—C22—C21	123.7 (4)
C7—C6—H6	119.3	C24—C23—C22	121.7 (5)
C8—C7—C11	116.7 (5)	C24—C23—H23	119.2
C8—C7—C6	124.9 (5)	C22—C23—H23	119.2
C11—C7—C6	118.4 (5)	C25—C24—C23	118.8 (5)
C9—C8—C7	120.3 (5)	C25—C24—H24	120.6
C9—C8—H8	119.9	C23—C24—H24	120.6
C7—C8—H8	119.9	C26—C25—C24	119.9 (5)
C8—C9—C10	119.6 (5)	C26—C25—H25	120.0
C8—C9—H9	120.2	C24—C25—H25	120.0
C10—C9—H9	120.2	C25—C26—C27	122.4 (5)
N2—C10—C9	122.5 (5)	C25—C26—H26	118.8
N2—C10—H10	118.7	C27—C26—H26	118.8
C9—C10—H10	118.7	C26—C27—C22	117.7 (5)
N2—C11—C7	123.2 (5)	C26—C27—C28	118.5 (4)
N2—C11—C12	116.4 (4)	C22—C27—C28	123.7 (4)
C7—C11—C12	120.3 (5)	C27—C28—H18A	109.5
N1—C12—C4	124.1 (4)	C27—C28—H28B	109.5
N1—C12—C11	116.1 (4)	H18A—C28—H28B	109.5
C4—C12—C11	119.8 (4)	C27—C28—H28C	109.5
O2—C13—O1	122.1 (5)	H18A—C28—H28C	109.5
O2—C13—C14	122.2 (5)	H28B—C28—H28C	109.5
